# Treatment of super oxide dismutase assay by a regression method

**DOI:** 10.1155/S146392468600007X

**Published:** 1986

**Authors:** Ph. Nirde

**Affiliations:** Centre d'immunologie & de biologie parasitaire Unité mixte INSERM U167 CNRS 624 Institute Pasteur, BP 245 Lille Cedex 59019 France


**Journal of Automatic Chemistry**/*Journal of Clinical Laboratory Automation*, **Volume 8, Number 1 (January–March 1986), pages 28–31**

### Treatment of super oxide dismutase assay by a regression method

**Ph. Nirde**

*Centre d'immunologie & de biologie parasitaire, Unité mixte INSERM U167, CNRS 624, Institute Pasteur, BP 245, 59019 Lille Cedex, France*

### Introduction

The superoxide dismutase (SOD, EC 1.15.1.1) is a widely distributed enzyme in all aerobic cells [1 and 2]. It is the principal protective enzyme against oxygen toxicity and acts by catalysing the removal of superoxide radical ($0_2^{-}\cdot$) [3 and 4]. The assay of SOD using Xanthine-Xanthine oxidase-Ferricytochrome C was described by McCord and Fridovich in 1969 [5]. Since, this spectroscopic method has been in use for SOD studies in both the medicine and biology.

The assay is based on the rate of reduction of ferricytochrome C measured spectrophotometrically, but this rate of reduction is not proportional to the amount of enzyme added. Therefore samples must be concentrated or diluted to obtain 50% of inhibition of the rate of reduction of ferricytochrome C: one enzyme unit [5]. So this method is not suitable for biologists working on scarce materials where storage of samples, to have enough material for an assay, may be prejudicial to the enzyme molecule.

In order to make the assay available for all samples, a minicomputer program has been developed. Because samples need not be concentrated nor diluted, this program provides a rapid and a useful calculation for the determination of the amount of SOD in samples, especially for biological extracts with low quantities. In addition, the sensitivity of the assay has been improved by a factor of 30 with this computer-aided approach.

### Hardware configuration

The WANG 2200 MVP model has a user memory of 45 K bytes. Only 14 K bytes are necessary for the resident program and the variables. This model has an integrated CRT/keyboard which displays 24 lines on screen, each 80 characters in length. The computer is equipped with a fast printer (2221W WANG) for hard copies and a hard disk unit for storing the program.

### Software

The WANG BASIC language allows 14 matrix operations. These operations are performed on numeric arrays according to the rules of linear algebra and can be used for the solution of systems of non-singular homogenous linear equations. The 'MAT INV' matrix statement causes matrix inversion by the Gauss-Jordan elimination method. A matrix can be inverted in a significantly shorter time than is possible with BASIC programs. Indeed, whereas other minicomputers store their program text exactly as it is entered by the programmer, the WANG BASIC interpreter converts into one-byte 'text atom' each word of the text program. Thus, the atomized lines permit faster program execution—lines are more compact and can be more rapidly scanned, for example, inverting a $7 \times 7$ matrix requires 0·20 s. Because no files are used for this logiciel, this program can be easily adapted to BASIC microsoft language.

### Mathematical expressions

*Rate of reduction of ferricytochrome C: least squares estimation*

The model, for calculating the rate of reduction of ferricytochrome C at a known enzyme amount, is:

$$y = a + b\,x \qquad (1)$$

where $y$ is the absorbance and $x$ the time when absorbance is set up. In the least squares estimation, the parameters $a$ and $b$ are chosen so that the value:

$$Q = \Sigma[y-(a + b\,x)]^2 \text{ is minimum.}$$

This condition occurs when the following partial derivatives are zeroing:

$$\frac{\delta Q}{\delta a} = \frac{\delta Q}{\delta b} = 0$$

$$\text{Thus, } \begin{cases} \dfrac{\delta Q}{\delta a} = 0 \\ \dfrac{\delta Q}{\delta b} = 0 \end{cases} \Longleftrightarrow \begin{cases} \Sigma(y - a - b.x) = 0 \\ \Sigma x.(y - a - b.x) = 0 \end{cases} \Longleftrightarrow$$

$$\begin{cases} \Sigma y - na - b\Sigma x = 0 \\ \Sigma y.x - a\Sigma x - b\Sigma x^2 = 0 \end{cases} \Longleftrightarrow \begin{cases} a = (b\Sigma x - \Sigma y)/n \\ b = \dfrac{\Sigma x.y - (\Sigma x.\Sigma y)/n}{\Sigma x^2 + (\Sigma x)^2/n} \end{cases}$$

*Relationship between rate of reduction of ferricytochrome C and enzyme amount*

The $p$-degree polynomial regression model used with one independent variable is:

$$y = a_0 + a_1\,x + a_2\,x^2 + \ldots\ldots + a_p\,x^p \qquad (2)$$

where $a_i$ is called the partial regression coefficient [6].

For each $y_i$, $x_i$ of $N$ data points, $p + 1$ sets of simultaneous linear equations can be written down:

$$\begin{array}{lllll}
\Sigma y = a_0 & N + a_1 & \Sigma x + \ldots\ldots + a_p & \Sigma x^p \\
\Sigma x y = a_0 & \Sigma x + a_1 & \Sigma x^2 + \ldots\ldots + a_p & \Sigma x^{p+1} \\
\Sigma x^2 y = a_0 & \Sigma x^2 + a_1 & \Sigma x^3 + \ldots\ldots + a_p & \Sigma x^{p+2} \\
\vdots & \vdots & \vdots \quad \vdots & \vdots \\
\Sigma x_p y = a_0 & \Sigma x_p + a_1 & \Sigma x^{p+1} + \ldots + a_p & \Sigma x^{2.p}
\end{array}$$

The numerical coefficient on the right side (i.e. $\Sigma x^j$) form a matrix $M$ which determinant is $D$. These coefficients involve only the $x$s.

$$M = \begin{vmatrix} N & \Sigma x & \ldots\ldots \Sigma x^p \\ \Sigma x & \Sigma x^2 & \ldots\ldots \Sigma x^{p+1} \\ \Sigma x^2 & \Sigma x^3 & \ldots\ldots \Sigma x^{p+2} \\ \vdots & \vdots \quad \ldots\ldots & \vdots \\ \Sigma x^p & \Sigma x^{p+1} & \ldots\ldots \Sigma x^{2.p} \end{vmatrix}$$

Those on the left also involved the $y$s. Resolution of such a system is mde by Cramer's method: the determinants of the $i$th partial regression coefficient, $D_i$, are:

$$D_0 = \begin{vmatrix} \Sigma y & \Sigma x & \Sigma x^p \\ \Sigma y.x & \Sigma x^2 & \ldots \Sigma x^{p+} \\ \Sigma y.x^2 & \Sigma x^3 & \ldots. \Sigma x^{p+} \\ \vdots & \vdots & \vdots \\ \Sigma y.x^p & \Sigma x^{p+1} & \ldots \Sigma x^{2*p} \end{vmatrix}, D_1 = \begin{vmatrix} N & \Sigma y & \ldots. \Sigma x^p \\ \Sigma x & \Sigma y.x & \ldots. \Sigma x^{p+1} \\ \Sigma x^2 & \Sigma y.x^2 & \ldots. \Sigma x^{p+2} \\ \vdots & \vdots & \vdots \\ \Sigma x^p & \Sigma y.x^p & \ldots. \Sigma x^{2*p} \end{vmatrix}$$

$$D_p = \begin{vmatrix} N & \Sigma x & \ldots. \Sigma y \\ \Sigma x & \Sigma x^2 & \ldots. \Sigma y.x \\ \Sigma x^2 & \Sigma x^3 & \ldots. \Sigma y.x^2 \\ \vdots & \vdots & \vdots \quad \vdots \\ \Sigma x^p & \Sigma x^{p+1} & \ldots. \Sigma y.x^p \end{vmatrix}$$

Thus, the partial coefficient regressions are:

$$a_0 = D_0/D,\ a_1 = D_1/D, \ldots\ldots, a_p = D_p/D$$

The expression of the polynome $y = P(X)$ is:

$$Y = x^0 .D_0/D + x^1 .D_1/D + x^2 .D_2/D + \ldots. + x^p .D_p/D$$

The root of such a polynomial regression is found by succesive iterations on the standard curve. In this case, two rates of reduction of cytochrome C from the standard are considered, including the rate of reduction of cytochrome C from the sample. If $X1$ and $X2$ are the corresponding enzyme amounts of the standard, these minimum and maximum values respectively enclose the unknown enzyme amount of the sample. For each iteration a half decrease of the segement ($X1$, $X2$) (i.e. $X3 = (X1, X2)/2$) is considered, which encloses the unknown enzyme value. If the calculated rate of reduction of cytochrome C from $X3$ is higher than the rate of reduction of cytochrome C from the sample, $X1 = X2$ is set as shown in figure 1. Another iteration is performed until the calculated rate of reduction is the same as the sample with an approximation of $10^{-4}$. This method converges rapidly.

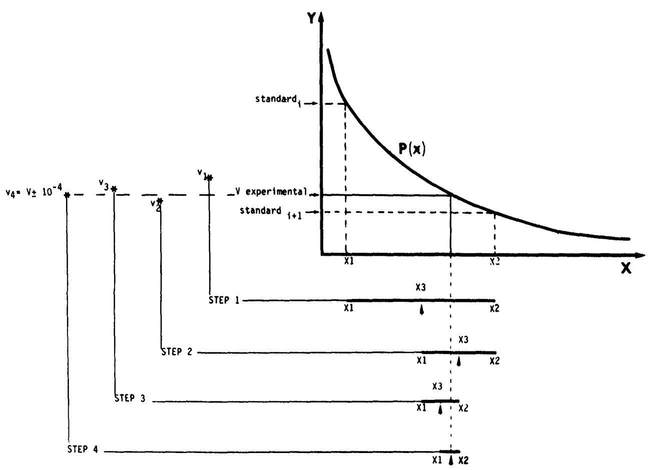

*Figure 1. Calculation of the polynomial's root by iterations.*

### Program description

*Standard curve*

As shown in figure 2, for a known enzyme concentration a set of experimental data points, $x_i$ (time) and $y_i$ (absorbance) are introduced. The coefficients $\Sigma x$, $\Sigma y$, $\Sigma x^2$ and $\Sigma x.y$ are calculated in a subroutine. Finally, the coefficient $b$ of equation 2 (i.e. the slope of the straight line) is computed; $b$ represents the rate of reduction of ferricytochrome C for a known enzyme amount.

Another subroutine computes the elements $\Sigma x^j$ and $\Sigma y.x^i$-used for polynomial regression. When only alphanumeric characters are keyed, the operator exits this part of the program and matrix and determinants are computed. The standard curve equation is then calculated and samples can be analysed.

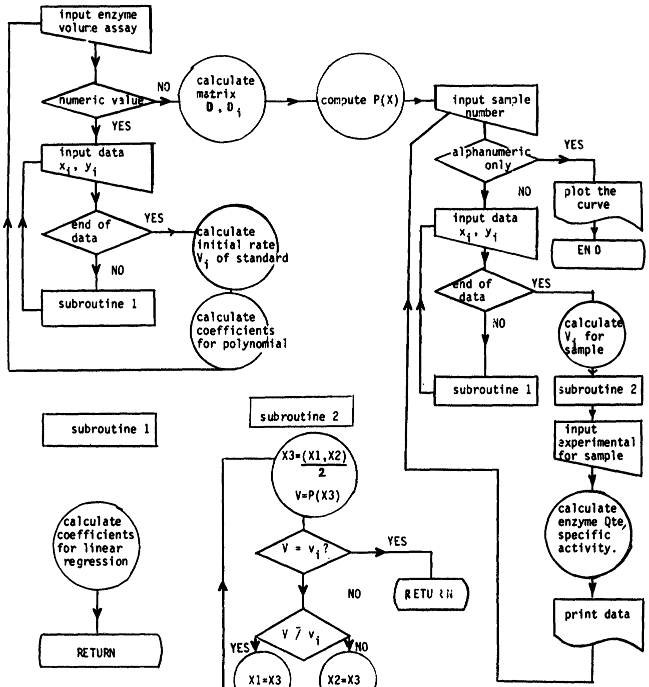

*Figure 2. Flowchart of polynomial fitting.*

*Sample analysis*

For samples, as for standard points, the rate of reduction of ferricytochrome C is calculated using a least squares estimation. This program allows the input of replicates by the FOR/NEXT loop. The mean value is calculated and the corresponding enzyme amount is computed by successive iterations in subroutine 2. Experimental parameters, such as initial extraction volume, assay volume and dilution, are integrated for the final calculation of the enzyme amount. Results are then printed. After the last sample is calculated the curve is plotted.

### Results

The polynomial curve is fitted with 11 experimental standards whose enzyme amounts are equidistant on the $x$-axis. Standards have been chosen so that the maximum and minimum rate of reduction of cytochrome C covers those of the samples. All experimental standard points are plotted on a curve (characters 'O')—see figure 3. In these experiments, samples (character '+') were a known dilution mixture of a standard solution. In this way we could verify that the calculated enzyme amount for samples was in good agreement with the real enzyme content. This was so throughout the whole range of 0–100% inhibition of the standard curve (table I).

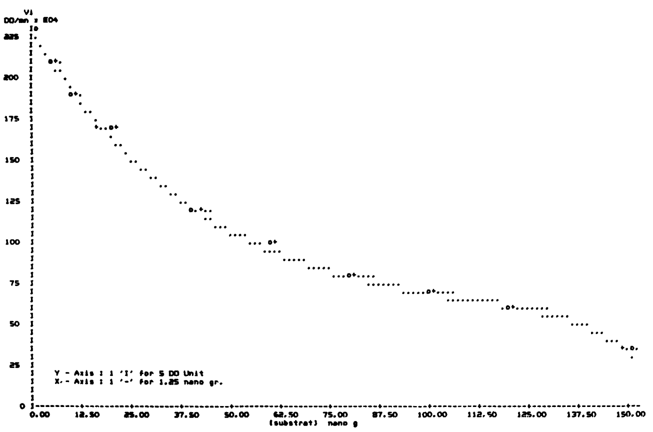

*Figure 3. Graph of polynomial model. Because using a typewriter instead of a plotter when experimental and calculated points have the same value all the points are plotted as '.' for theoretical curve, 'o' for standard points and '+' for samples.*

*Table 1. Results obtained using the mathematical model.*

December, 1984. Exper.: Polynomial adjustment.

SUPEROXIDE DISMUTASE SOLUTION USED : 1 micro g/ml - 21195 units/mg

| Volume | Initial speed | Qty SOD Sigma | Units Nb |
|---|---|---|---|
| 0.000 micro L. | 0.0234 D.O/mn | 0.00 nano g. | 0.0000 U. |
| 5.000 micro L. | 0.0214 D.O/mn | 5.00 nano g. | 0.1059 U. |
| 10.000 micro L. | 0.0194 D.O/mn | 10.00 nano g. | 0.2129 U. |
| 20.000 micro L. | 0.0170 D.O/mn | 20.00 nano g. | 0.4239 U. |
| 40.000 micro L. | 0.0122 D.O/mn | 40.00 nano g. | 0.8478 U. |
| 60.000 micro L. | 0.0100 D.O/mn | 60.00 nano g. | 1.2717 U. |
| 80.000 micro L. | 0.0082 D.O/mn | 80.00 nano g. | 1.6956 U. |
| 100.000 micro L. | 0.0072 D.O/mn | 100.00 nano g. | 2.1195 U. |
| 120.000 micro L. | 0.0064 D.O/mn | 120.00 nano g. | 2.5434 U. |
| 150.000 micro L. | 0.0036 D.O/mn | 150.00 nano g. | 3.1792 U. |

Yo = 233.3

| Sample Nb | Initial extract volume | Initial speed | % Inhibit. | Dilut. | Total Sigma S.O.D amount | Total enzyme units | specific activity |
|---|---|---|---|---|---|---|---|
| 0 µl | 0.25 ml | .02340 D.O/mn | 0 % | 1/ 1 | 0.00 nano g. | 0.0000 | |
| 1 µl | 0.25 ml | .02290 D.O/mn | 1 % | 1/ 1 | 1.09 nano g. | 0.0231 | 23182.03 U/mg |
| 2 µl | 0.25 ml | .02250 D.O/mn | 3 % | 1/ 1 | 2.10 nano g. | 0.0447 | 22354.10 U/mg |
| 5 µl | 0.25 ml | .02140 D.O/mn | 8 % | 1/ 1 | 5.01 nano g. | 0.1063 | 21277.79 U/mg |
| 10 µl | 0.25 ml | .01940 D.O/mn | 16 % | 1/ 1 | 10.78 nano g. | 0.2285 | 22850.85 U/mg |
| 15 µl | 0.25 ml | .01740 D.O/mn | 25 % | 1/ 1 | 17.34 nano g. | 0.3676 | 24506.71 U/mg |
| 20 µl | 0.25 ml | .01700 D.O/mn | 27 % | 1/ 1 | 20.00 nano g. | 0.4239 | 21195.00 U/mg |
| 40 µl | 0.25 ml | .01220 D.O/mn | 47 % | 1/ 1 | 41.48 nano g. | 0.8792 | 21981.53 U/mg |
| 60 µl | 0.25 ml | .01000 D.O/mn | 57 % | 1/ 1 | 60.00 nano g. | 1.2717 | 21195.00 U/mg |
| 80 µl | 0.25 ml | .00820 D.O/mn | 64 % | 1/ 1 | 80.62 nano g. | 1.7088 | 21360.58 U/mg |
| 100 µl | 0.25 ml | .00720 D.O/mn | 69 % | 1/ 1 | 100.15 nano g. | 2.1228 | 21228.11 U/mg |
| 120 µl | 0.25 ml | .00640 D.O/mn | 72 % | 1/ 1 | 120.00 nano g. | 2.5434 | 21195.00 U/mg |
| 150 µl | 0.25 ml | .00360 D.O/mn | 84 % | 1/ 1 | 149.98 nano g. | 3.1789 | 21192.93 U/mg |

From eight standard points onwards, the six degree polynomial gives satisfactory results for the SOD assay. The best results are obtained with 11 data points.

The reason for the development of a polynomial fitting for the SOD assay was the lack of methods available for studying biological material whose SOD content is very low. Whereas one unit was previously needed in the volume assay (50% of inhibition), 0·03 units are now sufficient with the polynomial fitting. Thus, the sensitivity of the enzymatic assay has been improved by a factor of 30. Assays can be performed on very little material and the program may be a useful tool for clinicians and researchers. Indeed, this treatment of SOD assay has allowed the detection of Cu/Zn SOD and Mn SOD in human *Schistosoma mansoni* [7]—so the biological role of these enzymes in *S. mansoni* can now be investigated. Furthermore, in tumor biology, low levels of SOD, particularly extra-cellular SOD (EC-SOD), have been recently reported, rarely in ostensibly normal human cell lines, but rather frequently in malignant cell lines [8]. Moreover, in clinical pneumology, modifications of the oxidant-antioxidant system within the human broncho-alveolar macrophages by intracytoplasmic mineral particules occur, in particular for SOD in coal-worker's pneumoconiosis and silicosis [9]. The assay system described should permit a more rapid and detailed investigations in these fields.

This mathematical model should be applied to research studies on SOD detection, purification, biological role, enzymatic regulation, as well as in a clinical context where large numbers of samples are routinely assayed.

### Discussion

The polynomial regression model is attractive because it is possible to approximate almost any function, such as the enzymatic assay for SOD. However, restrictions must be kept in mind when using such a mathematical model. The data on the $X$-axis occurs at equally spaced intervals, and in many cases the approximation is valid only in the region of the data, hence extrapolations are hazardous.

### Acknowledgments

The author thanks Professor J. P. Dessaint for valuable discussions and P. Fréssancourt for his skilled technical assistance.

### References

1. McCord, J. M. Keele, B. B. Jr. and Fridovitch, I., *Proceedings of the National Academy of Science,* **68**(5) (1971), 1024.
2. Fridovich, I., In *Free Radicals in Biology,* Ed Pryor, W. A. (Academic Press, New York, 1976), **1** 239.
3. Fridovich, I., McCord, J. M. and Michelson, A. M., In *Superoxide and Superoxide Dismutase,* Eds. Michelson, A. M., McCord, J. M. and Fridovich, I. (Academic Press, New York, 1977), p. 551.
4. Fridovich, I., *Science,* **201** (1978), 875.
5. McCord, J. M. and Fridovich, I., *Journal of Biological Chemistry,* **244** (22) (1969), 6049.
6. Freund, R. J. and Minton, P. D., In *Regression Methods,* Ed. Owen, D. B. (Marcel Dekker, Inc. New York, 1979), **30** 159.
7. Nirde, Ph., International Conference on 'Chemical communication and regulation of sexual reproduction, growth, and maturation in Schistosomes' (Baltimore, 1985).
8. Marklund, S. L., *Journal of Clinical Investigation,* **74** (1984), 1398.
9. Voisin, C., Wallaert, B., Aerts, C. and Grosbois, J. M., In *Third International Workshop on the in vitro Effects of Mineral Dusts* (Springer Verlag, Berlin, 1985).

### NEW JOURNAL

In February 1986, The Royal Society of Chemistry will launch a new international periodical entitled *Journal of Analytical Atomic Spectrometry (JAAS).* Published bimonthly, *JAAS* will contain original research papers, short papers, communications and letters concerned with the development and analytical application of atomic spectrometric techniques. It will also include comprehensive reviews on specific topics, general information and news of interest to analytical atomic spectroscopists including details of forthcoming conferences and book reviews.

An important feature of *JAAS* will be the inclusion of special reviews to be called Atomic Spectrometry Updates. These were formerly published in book form as part of Annual Reports on Analytical Atomic Spectroscopy, which will be discontinued following the introduction of *JAAS.* The review will cover a number of key areas including environmental and agricultural materials; chemical and food materials; instrumentation; industrial chemicals and metals; atomisation and excitation; and minerals and refactories. Successive issues of *JAAS* will review the entire range of topics previously covered by ARAAS to provide a unique current appreciation of developments in analytical atomic spectrometry.

The Editor is Mrs Judith Drew, with Dr J. M. Harnly as US Associate Editor. The Editorial Board includes:

Professor J. M. Ottaway (Strathclyde, UK)
Chairman
Dr M. S. Cresser (Aberdeen, UK)
Dr L. C. Ebdon (Plymouth, UK)
D. L. Miles (Wallingford, UK)
Dr B. L. Sharp (Aberdeen, UK)
Dr M. Thompson (London, UK)
Dr A. M. Ure (Aberdeen, UK)

and the Advisory Board:

Professor F. Adams (Antwerp, Belgium)
Professor R. M. Barnes (Amherst, MA, USA)
L Bezùr (Budapest, Hungary)
Professor R. F. Browner (Atlanta, GA, USA)
Professor L. de Galan (Delft, The Netherlands)
Dr J. B. Dawson (Leeds, UK)
Doz. Dr. sc. K. Dittrich (Leipzig, GDR)
Dr W. Frech (Umea, Sweden)
Dr A. Gray (Guildford, UK)
Professor S. Greenfield (Loughborough, UK)
Professor G. M. Hieftje (Bloomington, IN, USA)
Dr J. M. Mermet (Vernaison, France)
Professor Ni Zhe-ming (Beijing, China)
Dr N. Omenetto (Ispra, Varese, Italy)
Sir Alan Walsh (Victoria, Australia)
Dr B. Weiz (Uberlingen, FRG)
Professor T. S. West (Aberdeen, UK).

*The 1986 subscription (ISSN 0267-9477) is £165 (UK) $319 (USA) and £182 (rest of the World). Orders to Royal Society of Chemistry, Distribution Centre, Blackhorse Road, Letchworth, Hertfordshire SG6 1HN, UK.*

